# Borderline personality disorder symptom networks across adolescent and adult clinical samples: examining symptom centrality and replicability

**DOI:** 10.1017/S0033291721004931

**Published:** 2023-05

**Authors:** Jessica R. Peters, Michael L. Crowe, Theresa Morgan, Mark Zimmerman, Carla Sharp, Carlos M. Grilo, Charles A. Sanislow, M. Tracie Shea, Mary C. Zanarini, Thomas H. McGlashan, Leslie C. Morey, Andrew E. Skodol, Shirley Yen

**Affiliations:** 1 Alpert Medical School of Brown University, Providence, USA; 2 VA Boston Healthcare System, Boston, USA; 3 Butler Hospital, Alpert Medical School of Brown University, Providence, USA; 4 Rhode Island Hospital, Alpert Medical School of Brown University, Providence, USA; 5 University of Houston, Houston, USA; 6 Yale School of Medicine, New Haven, USA; 7 Wesleyan University, Middletown, USA; 8 McLean Hospital, Harvard Medical School, Boston, USA; 9 Texas A&M University, College Station, USA; 10 University of Arizona College of Medicine, Tucson, USA; 11 Beth Israel Deaconess Medical Center, Massachusetts Mental Health Center, Harvard Medical School, Boston, USA

**Keywords:** Adolescents, affective instability, borderline personality disorder, DSM-5 diagnosis, identity disturbance, network analysis, psychopathology

## Abstract

**Background:**

Numerous theories posit different core features to borderline personality disorder (BPD). Recent advances in network analysis provide a method of examining the relative centrality of BPD symptoms, as well as examine the replicability of findings across samples. Additionally, despite the increase in research supporting the validity of BPD in adolescents, clinicians are reluctant to diagnose BPD in adolescents. Establishing the replicability of the syndrome across adolescents and adults informs clinical practice and research. This study examined the stability of BPD symptom networks and centrality of symptoms across samples varying in age and clinical characteristics.

**Methods:**

Cross-sectional analyses of BPD symptoms from semi-structured diagnostic interviews from the Collaborative Longitudinal Study of Personality Disorders (CLPS), the Methods to Improve Diagnostic Assessment and Service (MIDAS) study, and an adolescent clinical sample. Network attributes, including edge (partial association) strength and node (symptom) expected influence, were compared.

**Results:**

The three networks were largely similar and strongly correlated. Affective instability and identity disturbance emerged as relatively central symptoms across the three samples, and relationship difficulties across adult networks. Differences in network attributes were more evident between networks varying both in age and in BPD symptom severity level.

**Conclusions:**

Findings highlight the relative importance of affective, identity, and relationship symptoms, consistent with several leading theories of BPD. The network structure of BPD symptoms appears generally replicable across multiple large samples including adolescents and adults, providing further support for the validity of the diagnosis across these developmental phases.

## Introduction

Borderline personality disorder (BPD) is a severe psychiatric disorder entailing intense, unstable affect; self-destructive, impulsive behavior; difficulties with interpersonal relationships; and unstable identity (American Psychiatric Association, 2013). A diagnosis of BPD can be met by the presence of any combination of at least five of the nine DSM diagnostic criteria, resulting in a highly heterogenous disorder with 256 possible combinations of symptoms. Numerous theories have arisen positing different core components of the disorder. Some highlight emotion dysregulation (Crowell, Beauchaine, & Linehan, 2009; Glenn & Klonsky, 2009; Linehan, 1993), including affective lability and components of impulsivity (Links, Heslegrave, & van Reekum, 1999), as central. Other theories focus on difficulties in interpersonal relationships (Gunderson, 2007) and understanding of the self (Kaufman & Meddaoui, 2021; Kerr, Finlayson-Short, McCutcheon, Beard, & Chanen, 2015) as hallmarks of BPD. Factor analysis of the nine symptoms found a three-factor grouping into the categories of affective dysregulation, behavioral disinhibition, and disturbed relatedness (Sanislow, Grilo, & McGlashan, 2000; Sanislow et al., 2002); however, these analytic approaches do not examine how these factors and symptoms within them interconnect within the disorder.

Network analysis has recently gained traction as a method to model psychopathology symptoms (Borsboom, 2017). In contrast to latent approaches that model symptoms as indicators of an underlying, single source of variance (dimensions), the network approach models symptoms as a reinforcing network of biological factors, emotions, cognitions, and behaviors. Symptoms, modeled as network ‘nodes,’ are assumed to cohere due to causal relationships between them (modeled as ‘edges’), rather than due to a single underlying cause. This approach may hold particular relevance for disorders such as BPD whose symptoms span a wide range of affect, cognition, and behavior that may emerge at different developmental points, differentially influence each other, and thus vary in how central their role is within the syndrome (Fried & Cramer, 2017).

In addition to modeling the expected influence of symptoms within networks, network structures can be compared across samples, allowing researchers to examine the replicability of symptom networks and specific network characteristics (Costantini et al., 2019; Fried et al., 2018). This facilitates comparisons across meaningful sample differences, such as developmental stages. Although the DSM permits diagnosing BPD in adolescents with marked and persistent symptoms of the disorder (American Psychiatric Association, 2013) and mounting evidence demonstrates the validity of the diagnosis in adolescents (Chanen, Sharp, & Hoffman, 2017), adolescent BPD remains a controversial topic (Miller, Muehlenkamp, & Jacobson, 2008), with many clinicians and researchers misinformed on the topic (Griffiths, 2011; Laurenssen, Hutsebaut, Feenstra, Van Busschbach, & Luyten, 2013), and adolescent BPD is thus underdiagnosed, undertreated, and understudied (Sharp & Fonagy, 2015).

Limited work to date has applied network approaches to BPD symptoms, all in adults. In one study of a large, non-clinical student sample, as well as a small (*N* = 96) clinical sample, affective lability was the most central self-reported BPD symptom across indices (Richetin, Preti, Costantini, & De Panfilis, 2017); these findings were replicated in a study of a large sample (*N* = 5221) of psychiatric patients (Peckham et al., 2020). Similarly, a study using a large sample of students and treatment-seeking community members modeled networks of self-reported BPD features (rather than diagnostic criteria), also finding a central role of emotion dysregulation, as well as interpersonal difficulties (Southward & Cheavens, 2018). A limitation across all of the network studies is that none use clinical diagnostic interviews to assess the BPD criteria modeled in networks, and the only study in a large clinical sample used a screening measure meant to detect *possible* symptoms with higher sensitivity (i.e. the McLean Screening Instrument for BPD), *v.* measures with higher specificity to determine symptom presence.

Only one study examined potential age effects on network structure, finding a stronger link between anger and unstable relationships and a weaker link between emptiness and suicide in participants aged >46 years compared to participants ⩽46 years old (Peckham et al., 2020). However, this study did not include adolescents and therefore cannot address issues of BPD network stability across this key developmental stage. In fact, no work published to date has examined BPD symptom networks in adolescent samples (clinical or otherwise) or compared networks across adolescent and adult samples. While prior work examined intercorrelations among BPD criteria in adolescents compared with adults (Becker et al., 1999), this study used small samples (Ns <50) and did not utilize methods like network analysis that can compare network features and centrality of specific nodes.

The goals of the present study were to estimate networks and centrality estimates of BPD symptoms, established through validated diagnostic interviews, and to estimate these networks across three large clinical samples of variable age and BPD symptom severity. We were interested in determining the degree of stability of network features across these different samples, as well as identifying divergent characteristics, with a particular focus on comparing the stability across adults and adolescents. We hypothesized that affective instability would be a central feature across all three networks, based on prior findings. We did not have a hypothesis about the exact degree of replicability of BPD network structure nor which features would diverge.

## Methods

### Participants

Data from three previously collected samples were analyzed for the present study. One sample is the baseline data from the Collaborative Longitudinal Study of Personality Disorders (CLPS), a longitudinal, multisite study examining the course of personality disorders, based out of four Northeastern US medical centers (Gunderson et al., 2000; Skodol et al., 2005). Participants (*N* = 733; 731 with complete BPD symptom data used) were recruited from clinics with advertisements targeting individuals with current or prior treatment history. Individuals meeting criteria for one of four personality disorders (BPD, schizotypal, obsessive-compulsive, and avoidant) or major depressive disorder with no more than 2 PD criteria for any PD (and less than 15 total) were eligible to participate. The majority of participants were female (64%) and White (67%), with a mean age of 32.50 years (s.d. = 8.11) and a range of 18–45 years.

A second adult sample was obtained via the Methods to Improve Diagnostic Assessment and Service (MIDAS) project, based out of Rhode Island Hospital (Zimmerman, 2003). Participants (*N* = 3800) were recruited through a community-based, outpatient psychiatry practice affiliated with an academic medical center, where research is integrated as part of a standardized intake process (Zimmerman, 2003). The practice does not restrict services based on presenting issue or diagnosis, and therefore patients represent a range of psychiatric concerns. A subsample of 3651 with complete BPD symptom data were used in the present study. The majority of the patients were female (60%) and White (87%), with a mean age of 38.81 years (s.d. = 13.35) and a range of 16–85 years old.

An adolescent sample (*N* = 1021) was recruited in the Houston area from inpatient psychiatric hospital settings (*n* = 872; 85%), through consecutive admissions [see prior publications for further details (Sharp et al., 2009)] and an additional healthy comparison group (*n* = 149; 15%) recruited through schools and community resources (Penner, McLaren, Leavitt, Akca, & Sharp, 2019). The majority of the patients were female (64%) and White (62%), with a mean age of 15.10 years (s.d. = 1.43) and an age range of 12–18 years.

### Measures

In the CLPS sample, BPD symptoms were assessed using the Diagnostic Interview for DSM-IV Personality Disorders (DIPD-IV; Zanarini, Frankenburg, Sickel, & Yong, 1996). The DIPD–IV is a semi-structured interview assessing each criterion of the DSM–IV PDs, which are rated on a 3-point scale (0 = *not present*; 1 = *present but of uncertain clinical significance*; 2 = *present and clinically significant*). In the CLPS sample, interrater reliability of the DIPD–IV (kappa) was 0.68 for BPD (Zanarini et al., 2000).

In the MIDAS sample, BPD symptoms were assessed with the BPD module of the Structured Interview for DSM-IV Personality (SIDP-IV; Pfohl, Blum, & Zimmerman, 1997). Symptoms are scored on a 4-point scale (0 = *not present or limited to rare isolated occasions*; 1 = *subthreshold: some evidence of the trait but it is not sufficiently pervasive or severe to consider the criterion*; 2 = *full threshold: criterion is clearly present but may be better accounted for by an Axis I disorder*; 3 = *full threshold: criterion is clearly present but is not better accounted for by an Axis I disorder*). Given that scores of 2 and 3 do not differentiate symptom severity and also reflect a distinction not assessed in the other measures used, these scores were collapsed into a single score (2) for the present study. Interrater reliability for BPD criteria was assessed in a subsample (*n* = 44), with a mean kappa of 0.71.

Adolescent BPD symptoms were assessed using the Childhood Interview for Borderline Personality Disorder (CI-BPD; Zanarini, 2003). The CI-BPD is a semi-structured interview developed specifically for use with children and adolescents to assess BPD and has shown strong psychometric properties in adolescents (Michonski, Sharp, Steinberg, & Zanarini, 2013; Sharp, Ha, Michonski, Venta, & Carbone, 2012). The interview was adapted from the BPD module of the DIPD-IV interview, described above. Symptoms are scored as *absent* (0), *probably present* (1), or *definitely present* (2). In the present sample, 10% of the interviews (*n* = 107) were coded by a second-rater for interrater reliability. Agreement on dichotomous BPD diagnosis between the two raters was substantial (*κ* = 0.74, *p* < 0.01).

### Analyses

Ordinal BPD symptom data (ratings from 0 to 2) were used to estimate partial Spearman correlation symptom networks. All individual networks were estimated using the *R* (R Core Team, 2020) for statistical computing *bootnet* (Epskamp, Borsboom, & Fried, 2018) package. Networks were estimated individually for each sample using an Extended Bayesian Information Criterion (Chen & Chen, 2008) (EBIC) graphical LASSO (Tibshirani, 1996) regularization. This regularization approach penalizes for complexity to minimize the estimation of spurious edges and return a more conservative network (Epskamp et al., 2018). A threshold was also added to each network to increase reliability by excluding edges of limited clinical significance.^1^ Nonparametric bootstrapping was used to estimate edge weight accuracy and centrality stability and the identified confidence intervals were used to test the strength/influence differences of edges and nodes within samples (Epskamp et al., 2018).

Individual network nodes were compared across samples on several metrics. Our primary metric of centrality is expected influence, which performs comparably or better than the strength centrality metric (Robinaugh, Millner, & McNally, 2016). The expected influence of a node is the sum of the edge weights connecting it to other nodes. Additionally, we also estimated and present node predictability, defined as the percentage of variability in a given node that is associated with other nodes in the network (Haslbeck & Waldorp, 2018). The higher a predictability score, the more that node can be accounted for by other internal network nodes, relative to variability independent from other nodes.

To examine differences between the networks, the expected influence of both individual nodes (symptoms) and strength of edges (connections between symptoms) were compared across the samples in pairwise tests using the *NetworkComparisonTest* (*NCT*) (Fried et al., 2018; Van Borkulo et al., 2016), corrected for multiple comparisons using Benjamini and Hochberg False Discovery Rate (Benjamini & Hochberg, 1995), consistent with prior network analyses of BPD (Peckham et al., 2020). While the NCT package has only been validated for Pearson partial correlation networks, the Pearson and Spearman correlation matrices were comparable for each sample (CLPS *r* = 0.99; MIDAS *r* = 0.98; Adolescent *r* = 0.99). R code for all analyses is provided in online Supplemental Materials.

## Results

### Sample descriptives

Symptom endorsement means and standard deviations in each sample, as well as patterns of significant differences between samples, are reported in Table 1. Across symptoms, the CLPS sample generally had the highest levels of endorsement, followed by the adolescent sample, followed by the MIDAS sample, with only one symptom not significantly different between each adjacent sample. Within the CLPS sample, 46% met the criteria for BPD (⩾5 of 9 symptoms rated 2), with *M* = 3.41 (s.d. = 2.70) criteria endorsed across the full sample. Within MIDAS, 10% met criteria, *M* = 1.74 (s.d. = 1.97), and within the adolescent sample, 26.2% met criteria, *M* = 2.78 (s.d. = 2.41).

**Table 1.** Means and standard deviations of BPD symptoms in three samples

	CLPS (*N* = 731)		MIDAS (*N* = 3651)		Adolescent (*N* = 1012)	
Symptom	*M*	s.d.	*M*	s.d.	*M*	s.d.
Intense anger	1.14^a^	0.87	0.67^b^	0.84	0.83^c^	0.89
Affective instability	1.23^a^	0.88	0.67^b^	0.86	0.99^c^	0.90
Feelings of emptiness	1.05^a^	0.89	0.64^b^	0.85	0.64^b^	0.84
Identity disturbance	0.88^a^	0.86	0.37^b^	0.72	0.65^c^	0.84
Stress-related paranoia or dissociation	0.84^a^	0.87	0.26^b^	0.62	0.78^a^	0.86
Efforts to avoid abandonment	0.75^a^	0.86	0.19^b^	0.52	0.49^c^	0.76
Suicidal or self-injurious behavior	0.64^a^	0.85	0.31^b^	0.65	1.16^c^	0.91
Self-damaging impulsivity	1.11^a^	0.90	0.67^b^	0.78	0.96^c^	0.95
Unstable relationships	1.02^a^	0.87	0.44^b^	0.75	0.62^c^	0.81

*Note*: Means with different superscripts are significantly different at *p* < 0.05. Symptoms were scored on the following three-point scales: CLPS (0 = not present, 1 = present but of uncertain clinical significance, 2 = present and clinically significant); MIDAS (0 = not present or limited to rare isolated occasions, 1 = subthreshold: some evidence of the trait but it is not sufficiently pervasive or severe to consider the criterion, 2 = full threshold: criterion is clearly present but may be better accounted for by an Axis I disorder or full threshold: criterion is clearly present and is not better accounted for by an Axis I disorder); Adolescent (0 = absent, 1 = probably present, 2 = definitely present).

### Independent network estimation

**Fig. 1.**
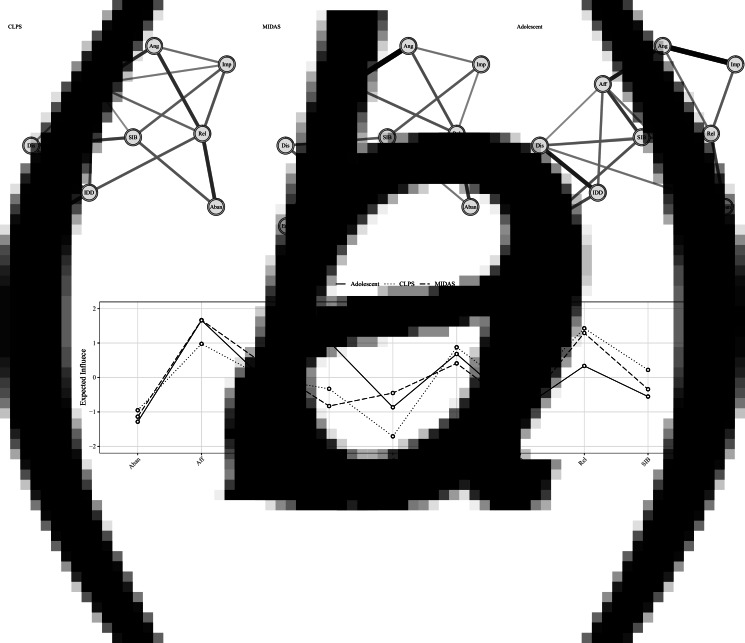
Individually estimated regularized partial Spearman correlation networks (*a*) and standardized expected influence (*b*) across three samples. Network edge thickness represents the degree of association. The gray area in the rings around the network nodes depicts predictability (the variance of a given node explained by all connected nodes). Ang, intense anger; Aff, affective instability; Emp, feelings of emptiness; IDD, identify disturbance; Dis, stress-related paranoia or dissociation; Abn, efforts to avoid abandonment; SIB, suicidal or self-injurious; Imp, self-damaging impulsivity; Rel, unstable relationships. All edges are positive. Edges with a magnitude of less than 0.1 are not displayed.

Individually estimated symptom network models and node influence estimates are presented in Fig. 1*a*. Network node locations are based on an average network and held constant across the samples to allow for easier visual comparisons. In the models, edge thickness represents edge weight; however, relative node *locations* in network models should not be used to infer centrality, which is instead presented in Fig. 1*b* graph. See online Supplementary Materials for tables providing all edge, expected influence, and predictability values for each network along with bootstrapped confidence intervals. In the CLPS network, predictability ranged from 0.09 (feelings of emptiness) to 0.28 (relationship instability). In the MIDAS network, predictability ranged from 0.17 (efforts to avoid abandonment) to 0.39 (affective instability). In the adolescent network, predictability ranged from 0.19 (efforts to avoid abandonment) to 0.36 (affective instability). The robustness of each network was estimated using the correlation stability (CS) coefficient (Epskamp et al., 2018).^2^ Network edges (CLPS CS = 0.53; MIDAS CS = 0.86; Adolescent CS = 0.68) and expected influence (CLPS CS = 0.41; MIDAS CS = 0.83; Adolescent CS = 0.48) were reliable.

**Fig. 2.**
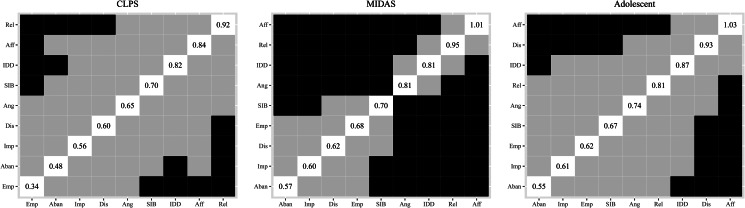
Expected influence centrality difference tests based on nanoparametric bootstrapping within each of the three samples. Black cell indicates a significant difference between the associated centrality estimates. The diagonal of each plot displays the observed expected influence for each of the nodes. Ang, intense anger; Aff, affective instability; Emp, feelings of emptiness; IDD, identify disturbance; Dis, stress-related paranoia or dissociation; Abn, efforts to avoid abandonment; SIB, suicidal or self-injurious; Imp, self-damaging impulsivity; Rel, unstable relationships.

Within each network, bootstrapped estimates were used to compare expected influence across nodes (see Fig. 2). Within the CLPS network, unstable relationships, affective instability, identity disturbance, and self-harming behaviors were all significantly greater in expected influence than at least one other node, and emptiness were significantly lower in expected influence than all four. In MIDAS, the nodes separated mostly into two groups, with affective instability, unstable relationships, identity disturbance, and intense anger all having significantly greater expected influence than most other nodes. In the Adolescent sample, affective instability was significantly stronger than most other symptoms, and stress-related paranoia or dissociation was stronger than four of the nine symptoms.

The overall networks demonstrated strong Spearman rank correlations between samples, with CLPS and MIDAS correlated at 0.78, CLPS and Adolescent at 0.55, and MIDAS and Adolescent at 0.46. Network comparison tests (see Table 2) demonstrated stronger global (across all nodes) expected influence within the CLPS network than the MIDAS network, and paranoia/dissociation having greater expected influence in the CLPS network and suicidal/self-injurious behaviors in MIDAS network. The CLPS and MIDAS networks had one difference in edge strength, with affective instability and anger more strongly associated in the MIDAS sample. The CLPS and adolescent networks had no significant differences in global expected influence or specific node expected influence differences and one difference in edge strength, with anger and impulsivity more strongly associated in the adolescent sample. The MIDAS and adolescent samples had the most significant differences, with stronger global expected influence within the adolescent network and with the dissociation/paranoia node having greater expected influence in adolescents. Seven (of 36) edges with strength differences. Affective instability demonstrated stronger associations with anger and relationships in MIDAS; other edge differences included a range of nodes and were stronger in the adolescent sample. Overall, these findings suggest that independently estimated network structures are largely similar across the samples, especially between the two adult samples (CLPS and MIDAS) and the two samples with higher levels of BPD pathology (CLPS and adolescent).

**Table 2.** Significant (with Benjamini–Hochberg corrections) network comparison results contrasting node strength and edge weights in each pair of BPD symptom networks

		Estimates by sample		*p* Value
		CLPS	MIDAS	
Global EI invariance		3.75	3.53	0.001
*Nodes*				
Paranoid		0.83	0.65	0.046
SIB		0.70	0.73	0.046
*Edges*				
Anger	Aff instability	0.21	0.36	0.029
		CLPS	Adol	
Global EI invariance		3.75	3.68	0.27
*Edges*				
Anger	Impulsivity	0.13	0.35	<0.001
		Adol	MIDAS	
Global EI invariance		3.68	3.53	0.011
*Nodes*				
Paranoid		0.93	0.65	<0.001
*Edges*				
Anger	Aff instability	0.25	0.36	0.019
Emptiness	Paranoid	0.17	0.06	0.018
Aff instability	Abandonment	0.14	0.04	0.018
Aff instability	SIB	0.21	0.05	<0.001
Emptiness	SIB	0.16	0.06	0.018
Anger	Impulsivity	0.35	0.12	<0.001
Paranoid	Relationships	0.16	0.04	0.018

## Discussion

Findings provide support for a largely replicable BPD network structure across three samples differing in age and levels of BPD pathology, including adolescent and adult samples. Networks were strongly correlated across the three samples, suggesting a largely uniform network structure for BPD. This is consistent with a prior finding examining BPD networks across age in adulthood that found few age-related changes (Peckham et al., 2020). While the network structures were strongly correlated, the MIDAS sample demonstrated a less interconnected network overall, relative to the CLPS and Adolescent samples, which may reflect the MIDAS study tapping a population with more diverse forms of psychopathology *v.* a higher rate of individuals endorsing the full BPD syndrome in the CLPS sample, which specifically recruited for personality disorders, and the Adolescent sample, which recruited from adolescent inpatient settings where BPD is relatively common.

Partially consistent with our hypothesis, affective instability emerged as a highly central symptom across all three networks; however, in each network, other symptoms were not distinctively less central, with identity disturbance similarly highly central across all three samples. Relationship instability emerged as relatively central as well in the two adult samples. The relative centrality of affective instability is consistent with past research identifying the trait as central to the BPD construct (Ebner-Priemer et al., 2015). The DSM-5 Section III Alternative Model of Personality Disorders (AMPD) includes emotional lability among the maladaptive traits diagnostic of BPD, which is a similar construct to affective instability (American Psychiatric Association, 2013). The observed centrality of relationship and identity disturbance is consistent with both the DSM-5 AMPD and ICD-11 (World Health Organization, 2018) characterizations of BPD, which identify instability of relationships and self-image as core functional impairments of the disorder.

Despite networks being largely similar, several significant differences in node centrality and edge weight emerged that may relate to differences between the samples. When samples only differed by one major characteristic (age or BPD pathology), network differences were minimal, but the two samples differing on both factors demonstrated the greatest number of divergent features. Unstable relationships had greater relative centrality in adult networks only, whereas dissociation/paranoia was among the most central nodes only in the adolescent network. These findings suggest the possibility that age and/or level of BPD pathology may influence the functioning of some symptoms within the BPD network. Relationship-related dysfunction, as well as fear of abandonment, may become more interlinked with other BPD symptoms as individuals age and develop more significant romantic relationships. The greater centrality of paranoid and dissociative symptoms in the adolescent sample than the MIDAS sample may reflect age differences, but also differences in severity of BPD pathology. For adolescents with severe psychopathology, stress from other BPD symptoms may be more likely to result in paranoia and dissociation or signal risk for other severe mental illness that may develop later in life. Similarly, the greater expected influence between anger and impulsive behavior in adolescents, relative to the two adult networks, may reflect heightened anger fueling more dysregulated behavior in younger individuals. Given prior findings of a stronger relationship between anger and relationship dysfunction within adults as they age (Peckham et al., 2020), it is also possible that the negative impact of maladaptive anger shifts as individuals develop, from primarily impulsive behavior in adolescence toward relationship impairment.

Replications across more samples with age and BPD severity differences are needed to test whether these sample characteristics are reliably linked to these differences in network characteristics or shared markers for varied prognoses. Future work should include multiple adolescent samples of varying levels of BPD, as well as samples of older adults, to better examine network constancy and changes over developmental course. Of note, none of these differences was sufficient to prevent strong correlations between the networks across all samples, suggesting an overall robust syndrome.

It is important to note that although network models are based on a causal theory, all analyses in the present manuscript are cross-sectional and correlational, and therefore causal inferences cannot be made from these findings. Rather, a key takeaway is that across each of these samples, generally similar patterns of associations between these symptoms emerge. These findings support a reproducible BPD syndrome, although given typically high rates of diagnostic co-occurrence with BPD, we acknowledge that this syndrome may be a broad indicator of personality pathology in general, and that more nuanced differences might emerge if symptoms of other disorders were also modeled (Sharp et al., 2015). To test hypotheses that these pathways may be causal, longitudinal data are needed to model network development and flux over time, particularly across key developmental time periods such as adolescence. In addition, variables that may validate causal explanations (e.g. neurobiological data) would be important in future work. Another potential limitation in the analytic approach includes the potential for the NCT procedure to have limited power, so the null findings should be conservatively considered. Future research with other samples may benefit from utilizing new analytic approaches to compare networks as these are developed and validated.

A strength of this study is the use of three clinical samples with differing levels of care and recruitment approaches; however, generalizability is limited given that the samples are from two geographic regions in the US with majority White participants. It is essential to extend this line of enquiry to samples representing all forms of diversity that may affect BPD network structure, with adequate numbers to test whether differences exist across those groups or based on environmental factors. Although a strength of the study was the use of validated clinical interviews to establish BPD criteria and the assessment tools had notable similarities across samples (clinician-administered, semi-structured, delivered by experienced and trained interviewers who were monitored to prevent drift), the exact interviews and rating scales varied slightly between samples, and this may have affected results. Using interviews with the same coding scales would be ideal in future work.

Overall, this study provides support for a replicable network of BPD symptoms across adolescent and adult samples of variable levels of BPD severity. Our findings support both conceptualizations of BPD that place either affective instability (Koenigsberg et al., 2002; Linehan, 1993) or identity disturbance (Fuchs, 2007; Jørgensen, 2010) as central to the disorder, consistent with prior network models of BPD (Richetin et al., 2017). These findings augment mounting evidence that BPD is a relatively generalizable and valid syndrome across adolescents and adults, indicating the need to continue efforts to understand the phenomenology of the disorder, as well as to expand both research studies into and clinical services for adolescent BPD.

## Notes

1 Elements of the precision matrix below  were removed (Epskamp, Cramer, Waldorp, Schmittmann, & Borsboom, 2012; Jankova & van de Geer, 2019)

2 The CS value indicates the maximum percentage of cases that can be dropped from the data while retaining, with 95% probability, a correlation of at least 0.7 between the statistics based on the original network and those computed with fewer randomly sampled cases. Values greater than 0.5 are desirable, values greater than 0.25 are acceptable (Epskamp et al., 2018).

## Supplementary material

For supplementary material accompanying this paper visit https://doi.org/10.1017/S0033291721004931. click here to view supplementary material
